# Social complexity as a driving force of gut microbiota exchange among conspecific hosts in non-human primates

**DOI:** 10.3389/fnint.2022.876849

**Published:** 2022-08-30

**Authors:** Braulio Pinacho-Guendulain, Augusto Jacobo Montiel-Castro, Gabriel Ramos-Fernández, Gustavo Pacheco-López

**Affiliations:** ^1^Doctorado en Ciencias Biológicas y de la Salud, Universidad Autónoma Metropolitana (UAM), Ciudad de México, Mexico; ^2^Department of Health Sciences, Metropolitan Autonomous University (UAM), Lerma, Mexico; ^3^Institute for Research on Applied Mathematics and Systems (IIMAS), National Autonomous University of Mexico (UNAM), Mexico City, Mexico; ^4^Center for Complexity Sciences, National Autonomous University of Mexico, Mexico City, Mexico

**Keywords:** social behavior, gut microbial communities, social microbiome, within-group microbial transmission, social brain hypothesis, microbiota, holobiont

## Abstract

The emergent concept of the *social microbiome* implies a view of a highly connected biological world, in which microbial interchange across organisms may be influenced by social and ecological connections occurring at different levels of biological organization. We explore this idea reviewing evidence of whether increasing social complexity in primate societies is associated with both higher diversity and greater similarity in the composition of the gut microbiota. By proposing a series of predictions regarding such relationship, we evaluate the existence of a link between gut microbiota and primate social behavior. Overall, we find that enough empirical evidence already supports these predictions. Nonetheless, we conclude that studies with the necessary, sufficient, explicit, and available evidence are still scarce. Therefore, we reflect on the benefit of founding future analyses on the utility of social complexity as a theoretical framework.

## Introduction

In less than 200 years, perceptions about the microbial world have sustained a transcendental shift: turning away from unsocial and disease-causing, to gregarious and beneficial across a series of niches and hosts, and as a link between all areas of life (Berg et al., 2020). This insight comes after the recognition that the majority of microorganisms are non-pathogenic (Casadevall and Pirofski, 2000) and that they may have a role in co-evolutionary relationships with their hosts, often resulting in mutually beneficial interactions in terms of fitness (Guerrero et al., 2013). In this sense several studies have contributed to our current understanding of how microbiota (including its genome—the microbiome) can influence the host’s health by disentangling its relationships host-related physiological, immunological, neurological, and developmental processes (Cryan and Dinan, 2012; Gerber, 2014; Stilling et al., 2014; Dinan and Cryan, 2017a,b; Rowland et al., 2018; Zheng et al., 2020). Microbiota is now recognized as a highly dynamic organ in space and time (Gerber, 2014; Donaldson et al., 2016; Uhr et al., 2019; Ji et al., 2020) modified by the dynamics of its microbial communities and numerous environmental forces, rather than solely influenced by host’s genetics and/or phylogeny (Archie and Theis, 2011; Gonzalez et al., 2011; Yatsunenko et al., 2012; Rothschild et al., 2018; Amato et al., 2019; Scepanovic et al., 2019).

Traditional approaches for the study of the relationship between the microbiome and social behavior have focused on the patterns and speed of pathogen transmission, however, contemporary approaches are based on a careful consideration of the extension, intensity, and ubiquity of microbial exchange between organisms and have supported the proposal that endosymbionts exchange could in fact be an “underappreciated” benefit of social interactions (Lombardo, 2008). Due to its role in inter-host transmission of microbes (Browne et al., 2017; Robinson et al., 2019; Sarkar et al., 2020), cumulative empirical studies suggest the influence of social behavior patterns on the characteristics of the microbiome and vice versa (Lombardo, 2008; Archie and Theis, 2011; Ezenwa et al., 2012; Montiel-Castro et al., 2013; Stilling et al., 2014; Archie and Tung, 2015; Johnson and Foster, 2018; Münger et al., 2018; Sylvia and Demas, 2018; Sherwin et al., 2019; Sarkar et al., 2020). These observations have led to the suggestion of the “*social microbiome*” concept (Sarkar et al., 2020). Defined as “the collective microbial metacommunity of an animal social group or social network” (Sarkar et al., 2020), this concept reflects the idea that the benefits provided by social life are highly intertwined with the paths of microbial transmission, which potentially arise as interactions amongst members of a given group. Thus, the composition of such microbial metacommunity may be influenced by social complexity, “in which individuals frequently interact in many different contexts with many different individuals, and often repeatedly interact with many of the same individuals in networks over time” (Freeberg et al., 2012).

Considering these definitions, our review is focused on unraveling the promising association between social complexity and the gut microbiota composition, in terms of diversity and similarity of microbial communities exhibited and shared among members of a social group. We start by proposing a general framework for exploring the possibility that gut microbiota interchange through social behavior could have important adaptive benefits to individuals within social groups. Then, we expand on the possibility that throughout their decision-making processes, subjects may also control over the selection of microbial communities (i.e., and not only genes or individuals) by expressing certain social behaviors and choice of particular social partners. Next, we use the framework of social complexity to propose several predictions describing how gut microbial diversity and/or similarity may vary in accordance with defining characteristics of social complexity in primate societies. Being highly gregarious organisms living in societies where an individual’s successful survival and reproduction depend on a network of complex social interactions (Maestripieri, 2010), primates are a suitable group to infer connections between social behavior and gut microbiota and, thus, we review primatological studies that offer evidence of the link between both variables.

Primate societies vary largely in size and composition, presumably due to socioecological conditions (Mitani et al., 2012; Strier, 2017), and are characterized by the establishment of strong and long-lasting social relationships (Seyfarth and Cheney, 2012a,b). As the number of these relationships increases with group size, social recognition and group management becomes more cognitively demanding processes, which has been evolutionary inferred from data on neocortex ratios (Dunbar, 1998). Hence, our next section tests the possibility of a quantitative association between social group size, neocortex ratio and gut microbial diversity across primate species. Notwithstanding the number of studies describing mechanisms by which microbiota interchange may influence individual fitness (Ezenwa et al., 2012), we suggest that the potential mechanisms that endosymbionts could employ to transfer between different hosts has not yet been thoroughly described. Thus, the final section of this review underlines the necessity that, to be a “collective metacommunity” (Sarkar et al., 2020), the social microbiome must either be horizontally transmitted across members of a group or environmentally acquired. Therefore, we suggest some of the potential biochemical and molecular mechanisms that gut bacteria could employ to tolerate the aerobic conditions of external environments and thus colonize other individuals’ internal environments.

### Linking sociality with primate gut microbiota: A novel driver of social evolution?

Overall, sociality is fundamental to regulate subjects’ health (Cacioppo and Cacioppo, 2014; Kappeler et al., 2015). In primates, including humans, a highly social integration, like within friendship network, may promote the prevalence of affiliative behaviors or positive habits in ways that encourage the host’s health (Umberson and Montez, 2010; Ostner and Schülke, 2018). In this regard, it has been observed an improvement on the diversity and richness of beneficial microbial communities (e.g., increase relative abundances of *Faecalibacterium, Akkermansia, Oscillospira*, and *Coprococcus*) in co-housed individuals with close and strong relationships, as in siblings and married couples (Brito et al., 2019; Dill-McFarland et al., 2019; Valles-Colomer et al., 2019; Johnson, 2020). Conversely, low levels of social integration are associated with an increased risk of premature death and suffering metabolic diseases and/or mental disorders (Umberson and Montez, 2010; Ostner and Schülke, 2018); under these conditions, gut microbiota composition undergoes an imbalance known as dysbiosis (Hooks and O’Malley, 2017) (e.g., decreasing relative abundances of beneficial microbes such as *Dialister*, *Corynebacterium* and *Coprococcus* with increments in abundances of non-beneficial ones like *Clostridium*, *Flavonifractor*, and *Oscillibacter*) (Valles-Colomer et al., 2019; Johnson, 2020), which appears to make hosts more vulnerable to opportunistic bacterial infections (Archie and Theis, 2011).

These two general arguments hide the possibility that social behavior allows horizontal transmission of microbes among individuals, which consequently implies that individual hosts serve as microbial patches connected via social interaction (Kuthyar et al., 2019; Robinson et al., 2019). In this sense, the degree of sociality could be conceived as an adaptive response mediating microbial transmission with an exchange of mutualistic and commensal endosymbionts among conspecifics and the physical environment (Archie and Theis, 2011; Ezenwa et al., 2016b; Miller et al., 2018; Münger et al., 2018; Kuthyar et al., 2019; Robinson et al., 2019).

Within this theoretical approach, more than three decades ago, Troyer (1984) proposed that health benefits obtained by belonging to a group could be explained by the social transmission of microbes. This idea was later expanded by Lombardo (2008) who suggested that animal social complexity could be more likely to develop and evolve when hosts must repeatedly obtain beneficial endosymbionts from conspecifics than when endosymbionts could be obtained directly from the environment. In agreement with previous suggestions and discussing the evolutionary role of specific social behaviors for promoting horizontal transmission of microbes in animal societies, we suggested quantifying the microbiota shared among conspecifics and use its inter-individual similarity as a measure of sociality (Montiel-Castro et al., 2013).

In addition to the possibility of microbial transmission involved in group living, Stilling et al. (2014) proposed considering the non-protein-coding regions, transcribed into RNA and with promising roles in neurodevelopmental processes, as part of an integrated model to understand the evolution of -human- social behavior. More recently, results of computational simulations driven by mathematical models in which natural selection acts on microbes harbored by interacting individuals show that microbes may affect the tendency of their hosts to cooperate or display paternal care behaviors (Lewin-Epstein et al., 2017; Gurevich et al., 2020; Lewin-Epstein and Hadany, 2020). Lastly, to fully capture the dynamics of host-microbial systems, ecological metacommunity theory has been expanded to include the feedback between the host-as-patch and its microbial communities as well as between the hosts and the species pool (Miller et al., 2018). From this ecological theory combined with principles of island biogeography theory, the term “social microbiome” has been recently proposed with the objective of facilitating the study of the influence of different microbial processes and their transmission across a host’s social networks, at multiple organizational levels (Sarkar et al., 2020).

Social behavior involves several emotional states (like anger or fear: Gothard and Hoffman, 2010) and cognitive processes (such as learning, memory, decision making, etc.) that occur in the brain, a highly energetically demanding organ (Dunbar and Shultz, 2017). From an evolutionarily perspective, the increase in brain size appears to be compensated by a reduction in the relative size of the gut in order to keep, presumably, the basal metabolic rate for the body at the typical level (Aiello and Wheeler, 1995). In addition, these organs are linked to each other through the bidirectional communication between the central nervous system and the enteric nervous system (gut–brain axis), which is mediated by neural, immune, and endocrine pathways (Montiel-Castro et al., 2013; Carabotti et al., 2015; Hyland and Cryan, 2016; Sherwin et al., 2019). This communication allows, for instance, for the emotional and cognitive centers of the brain to be tied-up with peripheral intestinal functions (Carabotti et al., 2015). Moreover, there is enough evidence of the importance of microbes in the gut-brain axis and, therefore, of its role as a component of individual and social behavior (Cryan and Dinan, 2012; Dinan et al., 2015; Sampson and Mazmanian, 2015; Parashar and Udayabanu, 2016). For instance, gut microbiota metabolizes complex lipids and polysaccharides (Rowland et al., 2018), and their metabolites (like short-chain fatty acids, bile acids, etc.) stimulate the production of gut hormones, influence thermogenesis, and act in the brain to regulate food intake (Pasquaretta et al., 2018; Cani et al., 2019).

Gut microbiota regulates genes linked to myelination, a determinant process for efficient transmission of nerve impulses in prefrontal cortex, underlying emotional regulation along with the amygdala, and facilitating memory storage, behavioral flexibility and attention (Hoban et al., 2016); processes that play a particularly important role in the management of social relationships (Aureli et al., 2012a). Furthermore, gut microbes may produce several neurotransmitters and hormones (Lyte, 2014; O’Mahony et al., 2015; Parashar and Udayabanu, 2016; Cussotto et al., 2018), which are the basis of the neuroendocrine control of complex social behaviors like attachment, social recognition, affiliation, and aggression (Klein and Nelson, 2010; McCall and Singer, 2012; Lieberwirth and Wang, 2014; Ziegler and Crockford, 2017). For instance, serotonin is synthesized by microbial members of the genera *Candida*, *Streptococcus*, *Escherichia*, and *Enterococcus*; dopamine and/or noradrenalin are generated by *Escherichia*, *Bacillus*, and *Saccharomyces*; and gamma-aminobutyric acid is produced by *Lactobacillus* and *Bifidobacterium* (Lyte, 2014; O’Mahony et al., 2015; Cussotto et al., 2018). These findings strongly suggest that gut microbes can interact with the neuroendocrine system, via the microbiota-gut-brain axis, to affect host behavior (Cussotto et al., 2018).

## Making choices in a social world: Conspecific and microbial partnerships

According to socioecological theory and the social brain hypothesis, group life is an adaptation to solve social problems while group size and social structure—the degree to which the sexes are related, the patterning of affiliative and agonistic interactions between individuals, etc. (Hinde, 1976)—seem to be constrained by individual capacity to process (i.e., recognize, remember, and manage) both social and ecological information to sustain and monitor social relationships (Cunningham and Janson, 2007; Dunbar and Shultz, 2017; Tremblay et al., 2017). Therefore, it is assumed that primates’ behavior is influenced by the characteristics of the socioecological environment in which they live (Janson, 2000; Thierry, 2008; Dunbar and Shultz, 2017) and thus, individuals must frequently make social (e.g., with whom to rest, forage, play, etcetera) and non-social (e.g., when and where to rest or forage) decisions to cooperate or compete with conspecifics, aiming to balance the costs and benefits associated with group living (Conradt and Roper, 2005; Barrett et al., 2007; Majolo and Huang, 2017).

Group life often involves conflicts of interest because individuals should make shared or unshared decisions for accessing to limited resources (mainly food and mates) (Koenig, 2002; Conradt and Roper, 2009) while coping with predation pressure and infection risk by steady exposure to pathogenic agents (Janson and Goldsmith, 1995; Altizer et al., 2003; Nunn and Altizer, 2006; Ezenwa et al., 2016a). Indeed, both direct (e.g., grooming) and indirect (e.g., overlapping range use) social behavior may increase the vulnerability to infectious and non-infectious diseases but also enhance individual resistance to pathogen infection (Altizer et al., 2003; Nunn and Altizer, 2006; Ezenwa et al., 2016a) and impact positively on individual reproductive success, longevity, and survival (Kappeler et al., 2015; Ostner and Schülke, 2018). Another interesting possibility regarding microbial exposure is that within-group cooperation and in-group microbial exchange actually limit the extent of an infection. Pathogens in a certain geographic area could be a selective pressure leading to assortative sociality, out-group avoidance, and limited dispersal (Fincher and Thornhill, 2008). These three factors could easily lead to enhanced within-group sociality, increasing cooperation with known group members and establishing strong behavioral frontiers limiting between-group microbial exchange. In other words, such purported frontiers could lead to preferential within-group social interactions and therefore, shape social structure. This pattern may be evident across different human cultures, where greater inter-individual microbial homogeneity has been reported, for example, in subjects with more collectively driven social values across cultures (Fincher et al., 2008), in tightly knit rural social structures in India (Das et al., 2018), and in people living traditionally in Nigeria (Ayeni et al., 2018). Moreover, it would appear as if the changes in lifestyle of indigenous communities upon exposure to industrialization and westernization imply losing crucial socio-ecological relationships with their environment (Schnorr et al., 2014). Also, the benefits of a richer, more diverse, and high metabolizing microbiota (Obregon-Tito et al., 2015) could be lost and replaced by one associated with diets consisting of high-sugar and-protein (Sánchez-Quinto et al., 2020). This could even include the unfortunate loss of beneficial bacteria carrying previously unknown functional antibiotic resistance genes harbored in the gut microbiota of isolated human populations such as the Yanomami in the Amazon (Clemente et al., 2015).

Kin selection–or inclusive fitness theory (Hamilton, 1964)—and multilevel—or group–selection are two complementary theories proposed in evolutionary biology for explaining the evolution of social behaviors (Marshall, 2011; Kramer and Meunier, 2016) regarding the ubiquitous nature of cooperation occurring both among simple microorganisms and within highly complex societies (Mehdiabadi et al., 2006; Kramer and Meunier, 2016). In this regard, kin selection theory claims that interacting individuals tend to help each other in accordance with the degree of relatedness between them, whereas multilevel selection theory proposes that selection acts both directly on individuals and at multiple levels of biological organization, including cells and/or groups of subjects (Hamilton, 1964; Kramer and Meunier, 2016). Both theories require positive assortment of (genetically) similar individuals for cooperative behaviors to evolve (Kramer and Meunier, 2016). Nevertheless, kinship plays a limited role in structuring social relationships (Chapais and Berman, 2004; Langergraber et al., 2009). Cooperative behaviors among unrelated individuals are usually based on reciprocity although the phenomenon is rare, so interactions between non-kin are likely to be maintained by mutualism or manipulative tactics involving coercion or inducement (Clutton-Brock, 2009). The evolution of cooperative behaviors should consider the strength of selection, the heritability of the group and individual level traits, and the genetic correlation between them (Goodnight, 2005). However, given different examples of adaptability provided by studies of symbiotic relationships with microorganisms (Li et al., 2008; Ezenwa et al., 2012; Cani et al., 2019; Gurevich et al., 2020), it is highly unlikely that natural selection could be applying any selective mechanisms only to genotypes or phenotypes of individuals.

Derived from theoretical approaches proposed to link host’s microbiota and sociality, an alternative and complementary reasoning to understand the evolution of social behavior considers that individuals throughout their decision-making processes, may control the expression of certain social behaviors for selecting microbes within other members of their social groups, and not only the individuals or the genes within them. Nonetheless, such selective control could result costly for individuals, who must consider the ubiquity and diversity of both “good” and “bad” microorganisms (Achtman and Wagner, 2008; Godon et al., 2016) and, consequently, taking into account that microbial exchange between members of a social network and its physical environment may be a continuous process (Sarkar et al., 2020). In this context, behavioral effects of the microbiota could readily arise as a by-product of natural selection on microorganisms within the host and natural selection on hosts to depend upon their symbionts (Johnson and Foster, 2018). These arguments open the questions of how individuals operate the purported microbial selection and whether microbes—or microbial communities—participate in their own selection by modifying the host’s behavior.

## Predicting variation in gut microbiota composition in relation to social complexity

The complexity of primate societies can be described based on the temporal variation of four distinct and interrelated components: (i) *social organization*, referring to group size, age-sex composition and the degree of spatial cohesion; (ii) *social structure*, describing the content, quality, and patterning of social relationships emerging from repeated interactions among conspecifics; (iii) *mating system*, involving the average number of males and females with whom each individual sustains mating interactions; and (iv) *care system*, providing information on who cares for dependent young as well as cooperative breeding in species where it plays a role (Kappeler and van Schaik, 2002; Kappeler, 2019). Conventionally, variation in group size is the most common measure of social complexity because it influences different aspects of the other components; however, all four components should be considered a coherent whole to understand the constraints and flexibility of primate social behavior (Kappeler et al., 2013) and define social complexity in a systematic manner (Kappeler, 2019).

According to Hinde’s framework, social systems—as a result of their complexity—have emergent properties that influence individual behavior and strategies (Hinde, 1976), which may imply either direct interaction among conspecifics (i.e., physical contact) or even mere spatial association (i.e., individuals in proximity or using the same places in different times). These social pathways may spread pathogenic agents causing infectious diseases among hosts (Altizer et al., 2003; Nunn and Altizer, 2006; Ezenwa et al., 2016b) as well as promote the exchange of mutualistic and commensal endosymbionts that may defend a given host from pathogens (Lombardo, 2008; Costello et al., 2012; Ezenwa et al., 2012). For instance, large aggregations of semi-social and eusocial insects are likely to attract pathogenic microorganisms, however, increments in group size are accompanied by increased antimicrobial strength (Turnbull et al., 2011).

Against this background, we expect that any social component could be linked to the structure of gut microbiota. In this sense, gut microbiota composition may vary accordingly to, for instance, spatiotemporal proximity patterns among conspecifics, with the number and duration of physical contacts because of the nature and quality of their multiple social interactions, or with the extent of overlap in their spatial range use. Following this reasoning, we formulate a series of predictions about how gut microbiota diversity and/or similarity may vary in relation to variation in social complexity and hopefully help understanding the potential key role of sociality on the horizontal microbial transmission between conspecific hosts.

### Social organization

Primates form temporal or permanent groups varying across three properties: (a) size, (b) age-sex composition, and (c) spatial cohesion (Mitani et al., 2012). Even in species categorized as ‘solitary’, individuals can associate sporadically (e.g., male and female may spend a relatively short time together and in close proximity during the mating season) or establish a tightly temporal aggregation (e.g., the offspring associating with their mother throughout the breeding period). Small or large groups occur in primates (Mitani et al., 2012). For example, northern gibbons (genus *Nomascus* and *Hoolock*) may form groups from three to seven individuals (Guan et al., 2018) while more than 300 individuals may be observed in the large gelada societies, *Theropithecus gelada* (Bergman et al., 2009).

#### Group size

**FIGURE 1:**
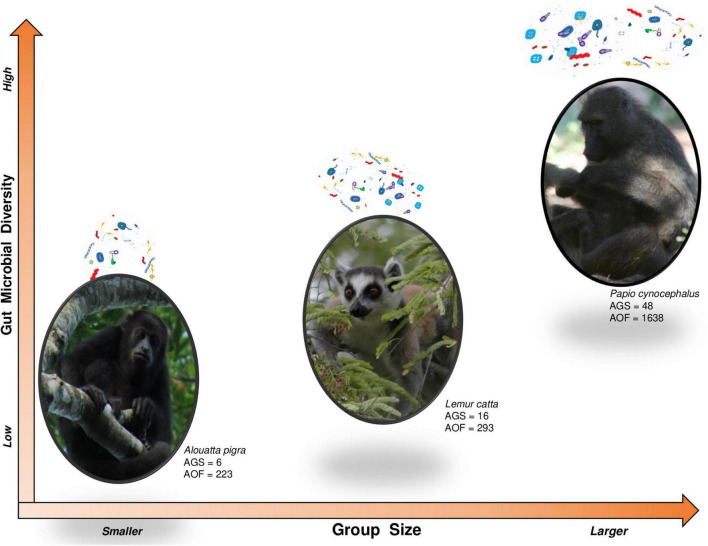
From ecology metacommunity theory, individuals serve as microbial patches pooling into the social microbiome. Therefore, we expect higher microbial diversity in larger groups, in turn limited by demographic processes, hosts’ properties, and environmental constraints. Furthermore, microbial similarity would be higher between individuals forming a stable, close and preferential association. Comparing data published for howler monkeys—*Alouatta pigra* (Amato et al., 2017) (credit: COBIUS AC – Image Database), ring-tailed lemurs—*Lemur catta* (Bennett et al., 2016) (credit: Frank Vassen, CC BY 2.0), and yellow baboons—*Papio cynocephalus* (Grieneisen et al., 2017) (credit: Augusto J. Montiel Castro), we observe a trend in microbial diversity (measured as average observed features—AOF) with regards to group size (average group size—AGS). In addition, it has been reported that microbial similarity was higher between female howler monkeys within proximity or contact than between distant females (Amato et al., 2017).

Diversity of vertebrate-associated microbes could scale up with social group size, as well as related to habitat size or animal body mass (Godon et al., 2016; Kieft and Nelson, 2010). However, gut microbiota composition may also be limited by demographic processes (i.e., birth, immigration, death, and dispersal of individuals: Shizuka and Johnson, 2020) as well as hosts properties (such as sex, age, health condition, social integration, reproductive status, dominance rank, among others) (Miller et al., 2018). Regardless of this, if we consider that each individual (host) essentially functions as a patch harboring specific microbial consortia (Gonzalez et al., 2011; Costello et al., 2012; Miller et al., 2018) and recognizing that these microbial communities are mostly divergent across hosts and highly dynamics over time (Costello et al., 2009; Caporaso et al., 2011; Lozupone et al., 2012), it is fair to assume, from an ecological perspective, some correspondence between group size and microbial pool size. Indeed, if microbiota exchange truly occurs among conspecifics and each individual contributes a distinct set of microbial communities to the social microbiome from that of others, we may expect large groups to have a higher microbial diversity than small groups (Figure 1). Also, if each host is able to harbor a limited set of microbial communities and a large social microbiome shows higher temporal dynamics, we may hypothesize that large groups undergo an overall decrease in microbial similarity amongst their members.

#### Age-sex composition

Regarding age-sex composition, primate groups are often diverse, and the number of males is often adjusted by the number of females (Lindenfors et al., 2004). Primate groups may contain one adult male and several adult females, as in gorillas (*Gorilla* spp.), several adult males and one adult female, as in Titi monkeys (*Callithrix kuhlii*), and multi adult males and multi adult females, such as in chimpanzees (*Pan* spp.) (Mitani et al., 2012). In all cases, adult males and adult females are accompanied by their infants and/or juveniles. In turn, given that biological maturation and aging are characterized, respectively, by the progressive development or decline of—gut—physiological functions (Lovat, 1996; Beunen et al., 2006) and that also females and males show sexually dimorphic patterns in energy and nutritional demands, which are often greater for females owing to gestation and lactation (Key and Ross, 1999; Markham and Gesquiere, 2017), the relative number of individuals in different age-sex classes may have important implications for individual energy gained (Markham and Gesquiere, 2017) and development due to anatomical, physiological, and behavioral differences (Dulac and Kimchi, 2007; Yang and Shah, 2014). Therefore, it should not be surprising to observe compositional changes in gut microbiota across the life cycle of males and females (Yatsunenko et al., 2012; Kostic et al., 2013; Dinan and Cryan, 2017b; Xu et al., 2019; Ojeda et al., 2021), which in turn could have an impact on the social microbiome. Thus, we propose that microbial diversity of a host first increases as the organism matures and then decreases as it senesces. In addition, we expect that females show a higher microbial diversity than males. In turn, we will expect that groups with several biologically mature females show a greater microbial similarity than groups with few mature females.

#### Spatial cohesion

Spatial cohesion may be measured by interindividual distances, allowing us to distinguish when one or more individuals separate (i.e., outside of visual range) or associate (i.e., in physical contact or proximity) with other conspecifics (Aureli et al., 2012b). These “fission and fusion” events imply changes in the number and identity of associates and are useful to define organizational subunits within the social group, such as subgroups or clans (Aureli et al., 2008, 2012b). Temporal variation of such events determines the degree of fission–fusion dynamics (Aureli et al., 2008), and consequently patterns of group cohesion. While spatial cohesion is a continuous variable, characteristic patterns can be exemplified by primate species, ranging from highly cohesive (e.g., represented by species with stable composition such as gorillas and howler monkeys) to highly fluid group (low cohesiveness) with variable composition, such as that observed in chimpanzees and spider monkeys (*Ateles* spp.) or those with a high variation in spatial cohesion but with relatively predictable compositions, like the multilevel geladas’ societies (Aureli et al., 2008).

As the intensity (frequency and duration) of proximity and social contacts promote pathogen infection (Altizer et al., 2003; Nunn et al., 2015; Ezenwa et al., 2016b) but perhaps also the mutualistic and commensal microbe transmission (Lombardo, 2008), association patterns and interindividual distances at the dyad and group level should be key determinants of microbial composition. We would expect that individual microbial diversity increases with the number of social partners in contact and close proximity networks, such that highly integrated individuals into these networks would have higher microbial diversity than individuals with low integration into association and proximity networks. Moreover, microbial similarity should be higher between individuals forming stable, close and preferential associations compared to that found in randomly- or distantly associated individuals.

As fission–fusion dynamics create opportunities for individuals to interact differentially with other subjects and their environment, microbial composition at the group level should presumably vary in concert to the degree of fission–fusion dynamics observed in a particular species and/or social group. Our prediction would be that greater microbial diversity would be a characteristic of highly fluid societies—where the variation in strength of association between group members is greater—compared that found in highly cohesive groups, where there is less variation in strength of association. Also, microbial similarity would be overall higher in cohesive groups, in which inter-individual distances are often relatively shorter, than in highly fluid groups, commonly showing high variation in inter-individual distances.

### Social structure

**FIGURE 2:**
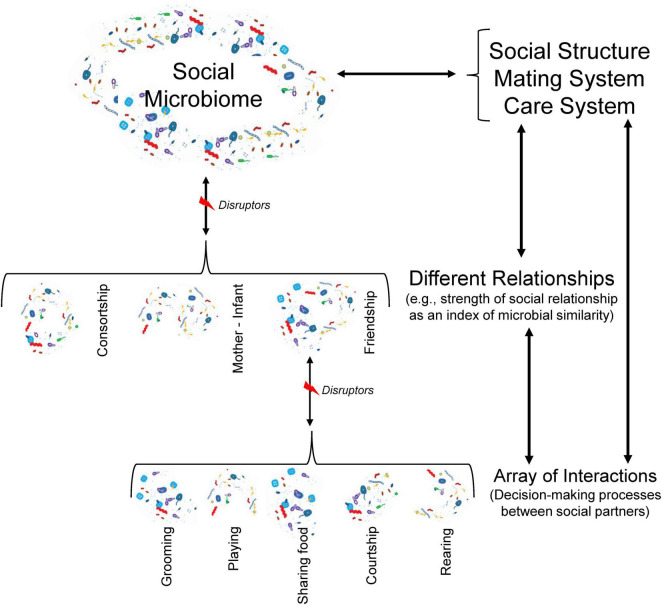
Individuals as microbial patches are connected by interactions, which result from a decision-making process (e.g., to whom care, with whom play or share food). Different microbes (in terms of quality and quantity) may be transmitted between partners in each kind of interaction. It is likely that many gut microbes could be exchanged during interactions involving mouth-anogenital contact. The nature (social, mating, and rearing contexts) and patterning (frequency and duration) of interactions determine the kind and strength of a relationship between two individuals (e.g., friendship, consort, etc.), which has been associated with microbial similarity (Montiel-Castro et al., 2013). This microbial exchange across distinct levels (interactions, relationships, and social components), however, may be disrupted by removal and/or social integration of individuals (such as juveniles or immigrants) into the social network, hosts’ properties (such as age, sex, health condition), environmental conditions (like humidity, temperature), and survival and colonization strategies of gut microbes. Patterns of different relationships define the social structure and mating and care systems, which influence the social microbiome. As microbiota-gut-brain axis suggests a bidirectional communication between enteric and nervous system (Hyland and Cryan, 2016), we will expect the social microbiome, in turn, influences different levels of social complexity of primate groups. In this figure, we also highlight the reciprocal influence among the three levels of social structure.

The nature and patterning of social interactions contribute more to interspecific variation in social complexity in species capable of individual recognition and repeated interactions (Thierry, 2013; Kappeler, 2019). This is particularly important in primate societies, where individuals rarely interact at random, establishing distinct types of relationships with different individuals, often leading to differentiated relationships (Silk, 2007b; Seyfarth and Cheney, 2012b) that may influence their competitive success and reproductive performance (Silk, 2007a,b). Owing to the social structure determines, for instance, the frequency and duration of—spatiotemporal—contact and proximity interactions and the strength of social relationships, it should also provide a major “blueprint” within microbial transmission at the group level, describing the social pathways through which microbes could be exchanged among conspecifics (Figure 2). In this regard, one study on a large cohort of indigenous people from Fiji suggests that strong intra-familial and between spouses’ microbial transmission patterns occurred and that, compared to men, women harbored strains more closely related to their familial and social contacts (Brito et al., 2019). The above suggests the prediction that for species with matrilines and strong male dispersal, due to sustained intergenerational vertical transmission, particular sets of microbial communities and their health-related benefits may characterize specific matrilines, and thus reflect some degree of similarity based on kinship. This avenue provides a novel explanation to understand why high-ranking females, within matrilineal dominance hierarchies such as in baboons and macaques, are prone to mature at earlier ages, grow faster, have shorter interbirth intervals, produce healthier infants, and have higher lifetime fitness than low-ranking females (Sapolsky, 2005; Silk, 2007b). On the other hand, where females disperse, e.g., as in chimpanzees, one would expect that in any case, the patterns and structure of kin-related male social relationships would more likely reflect any possible inter-individual microbial similarity. Therefore, as mentioned elsewhere, such the available or preferred social world could in turn restrict the available range of microorganisms than an individual of a given hierarchy can access, or not, at any given time (Sarkar et al., 2020).

Ecological models of social relationships often overemphasize competition and agonism while overlooking cooperative and affiliative behaviors (Sterck et al., 1997; Sussman et al., 2005; Koenig et al., 2013). However, most of primate social interactions are affiliative and, thus form the basis of social relationships (Sussman et al., 2005). Also, affiliation, cooperation, and social tolerance among conspecifics serve a crucial role in sustaining alliances, integrity of friendship network, protection from predators, cooperative infant care, and access to information and limited resources (Sterck et al., 1997; Sussman et al., 2005; Silk et al., 2013). In addition, the kind and strength of social relationships are determined by the frequency, duration and the context in which social interactions occur (Hinde, 1976; Silk et al., 2013). Then, if we assume relationships as social investments from which individuals may derive an associated benefit with microbial exchange, we could expect that individuals with high social integration (i.e., individuals interacting with several social partners across different contexts) show higher microbial diversity than individuals with low social integration (i.e., individuals interacting with few social partners across few contexts). Also, two individuals (i.e., a given dyad) interacting frequently and in a predictable and friendly way should have both stronger social relationships and concomitantly, a greater microbial similarity than two individuals with weak social relationships.

As a relevant factor influencing the dynamics of the social structure, we must also take into account the effects that demographic processes play as an influence on the patterns of social relationships among conspecifics (Shizuka and Johnson, 2020). For instance, death or dispersal can can have direct effects on the social structure, e.g., by removing an individual or even all its social connections (leading to the removal of entire microbial consortia), and indirect effects, e.g., by prompting changes in the distribution of social connections between the remaining individuals in a group. On the other hand, recruitment and integration of juveniles or immigrants into existing social networks are critical to the emergence and persistence of social network structure (Shizuka and Johnson, 2020). Derived from individual movements across groups (i.e., dispersal) or due to permanent fission events, a group’s social dynamics may be modified, affecting group members’ gut microbial communities (i.e., social microbiome). In chimpanzees, immigrants into a community harbor distinct taxonomically microbial phylotypes than resident individuals, suggesting that subjects retain hallmarks of their previous [social] gut microbial communities, even for extended periods (Degnan et al., 2012), perhaps increasing the gut microbial diversity of their new social group. Similar patterns were found in baboons, in which the longer an immigrant male had lived in a given social group, the more closely his gut microbiome resembled those of the group’s resident males (Grieneisen et al., 2017). This observation suggests that even when retaining microbial communities of its previous social group, the time length of an immigrant’s membership into its current social group can predict its similarity to the group’s social microbiome.

### Mating system

Traditionally, four categories of mating systems are recognized based on the number of mates: monogamy, polygyny, polyandry, and promiscuity. However, evidence of extra-pair copulations and of the variation in promiscuity is also abundant across primates (Kappeler, 2012). A first point of interest is that females are not expected to mate randomly if there is variation in male quality. For instance, if males do not provide paternal care, females often choose mates according to qualities gathered of visual and odor signals or clues (e.g., territory quality, health condition, body and testicle size, rank position, etc.) (Winternitz and Abbate, 2015). Immunogenetic competence is a highly adaptive trait in this context, one that can presumably be detected via olfaction in mammals (Penn, 2002; Winternitz and Abbate, 2015).

In insects, gut microbiota alters the scent of an individual, which likely affects processes related to mate choice and nestmate recognition (Lizé et al., 2013). On the other hand, in primates, a comparative analysis revealed host-specific vaginal microbiota and showed correlations between vaginal microbiota diversity and factors related to host sexuality, including female promiscuity, baculum length, gestation time, mating group size and neonatal birth weight (Yildirim et al., 2014). Promiscuity has also been tested to impact positively the bacterial communities’ diversity of reproductive conducts like cloaca and vagina in lizards (White et al., 2011) and rats (MacManes, 2011), respectively. Furthermore, in promiscuous primates, females can form temporary consortships or long-term friendships, develop sexual swellings, or produce olfactory signals of imminent ovulation (Kappeler, 2012) that seem to promote the precopulatory courtship behaviors like genital inspection.

As mating strategies to gain access to sexual partners often involve different patterns of proximity and mouth to anogenital contact, their variation may produce microbiome changes. Therefore, in accordance with the potential role of mating systems in shaping patterns of reproductive microbiome diversity (see Figure 3 in Rowe et al., 2020), we expect that the number of sexual partners and frequency of precopulatory courtship behaviors influence the composition of gut microbiota. In this sense, we would expect that individuals who frequently engage in precopulatory behaviors to mate with multiple sexual partners show a greater microbial diversity than individuals who mate with one or a few sexual partners. Accordingly, it seems reasonable to assume that microbial similarity would be diluted with the relative number of sexual partners of each individual.

**FIGURE 3:**
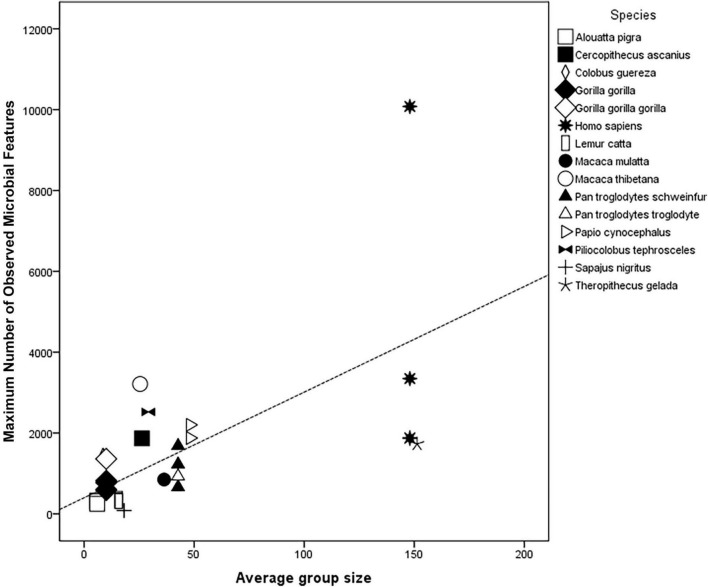
Scatterplot for regression results of species’ specific Average group size for 15 primate species (including *Homo sapiens*) as predictor of their Maximum number of observed microbial features (OTUs or ASVs); *R*^2^ = 0.42; Adj *R*^2^ = 0.40; *F*(3,30) = 20.7, *P* < 0.001; *N* = 31.

### Care system

Among primates, rearing strategies vary greatly, from exclusive maternal care to biparental care with significant male contribution and cooperative and communal breeding (Mitani et al., 2012). Direct male care is seen in approximately 40% of genera (Muller and Thompson, 2012). For instance, in Titi monkeys, males regularly play with, groom, and share food with infants (Fernandez-Duque et al., 2012) and, in captivity, infants develop a preference for their fathers over their mothers, as assayed by a stronger pituitary-adrenal stress response when they are separated from their fathers rather than their mothers (Hoffman et al., 1995). Whilst promiscuous mating is expected to reduce paternity certainty, in several species of baboons and macaques, males commonly carry, cuddle, play with, and protect infants (Muller and Thompson, 2012), opening an opportunity for microbiota interchange amongst parent-offspring dyads. A similar possibility could be suggested for species with important degrees of alloparenting (non-maternal care of infants), and it can be performed by relatives and non-relatives alike, helping to accelerate infants’ development and reduce their vulnerability to predation or infanticide (Lonsdorf and Ross, 2012).

Siblings may play particularly important roles in infant behavioral development and socialization, as they are likely to be frequent social partners (Lonsdorf and Ross, 2012). Allocare then, includes infant carrying and transport, guarding, food sharing, grooming, play, and nursing, are all behaviors that would make the exchange of microbial life more likely given the intensity and frequency of social interactions. Cooperative breeding, which is conspicuous among Callitrichines (Fernandez-Duque et al., 2012), occurs when mother and offspring receive extensive care from non-mothers—that is, males and non-reproductive females—whereas communal breeding refers to situations where mothers mutually provide allonursing and perhaps other forms of care to each other’s offspring (Lonsdorf and Ross, 2012). These fine patterns of social interactions among immature and mature individuals could play an important role in the development of the immune system and microbiota composition for infants, particularly in large groups where infants’ development is slow (Borries et al., 2008). In this sense, gut microbial communities develop and grow more specific with biological maturation in infants (this lasts at least three years after birth in humans: Kostic et al., 2013), which could be obscuring their microbial diversity and similarity patterns.

## Empirical evidence associating social complexity with gut microbiota composition

### Social organization

Overall, group membership seems to be a key factor of clustering when intraspecies comparisons of gut microbiota composition were carried out among individuals belonging to different social groups (Tung et al., 2015). Results in this sense have been observed in rhesus monkeys, howler monkeys, chimpanzees, ring-tailed lemurs, red-bellied lemurs, yellow baboons and colobus monkeys (Degnan et al., 2012; Tung et al., 2015; Bennett et al., 2016; Amaral et al., 2017; Amato et al., 2017; Grieneisen et al., 2017; Perofsky et al., 2017; Springer et al., 2017; Raulo et al., 2018; Goodfellow et al., 2019; Wikberg et al., 2020) among others. Nonetheless, the influence of group membership on microbiota composition seems to vary widely and relatively rapidly. For example, social group membership explained from 35 to 58% of the variance in gut microbiota composition of wild Verreaux’s sifakas and it remained as a significant predictor after controlling for genetic relatedness among subjects (Perofsky et al., 2017; Springer et al., 2017). On the other hand, in arboreal groups of white-faced capuchins, group membership had a minor impact on the gut microbiota, explaining only 6% of its variation, which may be related to the common large overlap in home ranges and shared use of the same water sources during dry season (Orkin et al., 2019).

The same could be said for chimpanzees (Degnan et al., 2012), for which a high fluidity of social relationships could obscure such a pattern. Nevertheless, homogenization of gut microbiota may occur rapidly via the formation of new groups. In young rhesus monkeys housed indoors under controlled environmental conditions, the formation of new and smaller groups was associated with significant shifts in the gut microbiota, with microbial convergence occurring only two weeks after new monkeys joined previously established social groups (Amaral et al., 2017). Altogether, we propose that gut microbiota differentiation found between social groups could be an effect of host behavior adapting to given socioecological conditions, including particular environmental and socio- microbial communities. In this regard, one further study found that while microbial communities were similar across individuals before a permanent group-fission, the microbial signatures of the two resulting groups of colobus monkeys were significantly different nine months after such fission event (Goodfellow et al., 2019). Changes in groups’ kin composition did not explain this pattern and the authors were unable to discern whether this divergence was due to the resulting groups having separate home ranges with different food resources or due to changes in the social network derived from the group fission (Goodfellow et al., 2019).

#### Group size

Until now, there is scarce and unclear evidence supporting our predictions concerning how gut microbiota composition may vary according to group size. Nonetheless, Greineisen and colleagues compared gut microbial communities between two baboon groups (27 vs. 51 individuals) finding that individuals living in the larger social group exhibited higher gut microbial diversity than individuals in the smaller social group (Grieneisen et al., 2017). Also, Raulo and colleagues compared alpha diversity of gut microbiota among eight family groups of red-bellied lemurs finding no correlation between group size and alpha diversity (Raulo et al., 2018). Authors note, however, that their ability to infer group size effects is limited due to the reduced number of social groups or due to small variation between group sizes. Additionally, gut microbiota composition of geladas was more similar among members of smaller organizational subunits (i.e., one-male units) compared to that found in larger subunits (i.e., clans or troops). This result supports our prediction if we consider the usual size variations of organizational subunits in multilevel societies. Nevertheless, this effect may also be related to the relatively stable age-sex composition or the short interindividual distances found in one-male units, even after splitting or joining other one-male units (Schreier and Swedell, 2012). In chimpanzees, gut microbial communities were more homogeneous when individuals spent more time together in large groups than when they spent more time alone or associated in small groups (Moeller et al., 2016).

### Age-sex composition

We predict that the relationship between age-sex composition of social groups and microbiome depends on the relative number of individuals in different age-sex classes, and that the microbiota composition of a host may be influenced by its biological maturation, aging and sex. Biological maturation and aging are routinely placed in the context of chronological age (Beunen et al., 2006). Under this assumption, humans show a great variation and relatively low diversity in their microbiota during infancy but become more diverse into adulthood (Kostic et al., 2013; Odamaki et al., 2016; Nagpal et al., 2018; de la Cuesta-Zuluaga et al., 2019; Xu et al., 2019; Radjabzadeh et al., 2020). The microbial alpha diversity continues increasing until the twenties (Odamaki et al., 2016) or forties (de la Cuesta-Zuluaga et al., 2019) when it stabilizes. In concordance, adult individuals of gorillas and gibbons showed greater microbial diversity than their infant and juvenile counterparts (Jia et al., 2018; Pafčo et al., 2019).

Aging in humans has been associated with a reduction in microbial diversity (Kostic et al., 2013; Nagpal et al., 2018; de la Cuesta-Zuluaga et al., 2019; Xu et al., 2019) but healthy aging often correlates with an increased or sustained microbial diversity (Odamaki et al., 2016; Dinan and Cryan, 2017b; Nagpal et al., 2018; Ojeda et al., 2021). On the other hand, sex also plays a pivotal role in the human microbiota. In this sense, women harbor a higher microbial alpha diversity than men (de la Cuesta-Zuluaga et al., 2019), and their diversity increases during pregnancy (Kostic et al., 2013; Radjabzadeh et al., 2020). Conversely, this sex-based microbiota difference was less evident in some non-human primates (Jia et al., 2018; Pafčo et al., 2019). Additionally, gut microbial communities were more similar among females than males in chimpanzees, howler and snub-nosed monkeys (Degnan et al., 2012; Amato et al., 2017; Liu et al., 2018). Overall, these individual patterns could influence the social microbiome. Nevertheless, there are still few comparative studies across a wide array of social groups for exploring the influence of age- and/or sex-biased group composition on the gut microbiota profiles.

#### Spatial cohesion

Evidence of the influence of interindividual distances (a measure of spatial cohesion) on gut microbiota composition is limited and controversial. In black howler monkeys, time spent in social contact had no relationship with gut microbiota similarity between individuals within a given social group, controlling for kinship. However, there was a marginally significant trend for individuals that spent more time in close proximity (0–1 m) to possess more similar gut microbiota. This pattern was driven by adult female dyads, which generally spend more time in social contact than adult male-adult male dyads or adult male-adult female dyads. Then, it is likely that the sex of individuals is obscuring the effect of spatial cohesion on gut microbiota similarity. When the analysis included adult female-adult female dyads only, gut microbial communities were more similar when females spent more time both in contact and in proximity (Amato et al., 2017). This result supports our prediction but more spatial data across groups is needed for its validation and also to evaluate whether variation in spatial cohesion patterns is associated with microbial diversity.

### Social structure

Current research into the mammalian microbiome suggests the possibility that the social microbiome may be a good proxy for species’ social structure (Sarkar et al., 2020). We can observe that in a similar way as primate species differ in their social structure (*sensu* Hinde, 1976) there are species’ level differences in terms of their gut microbial communities, with significant interspecies dissimilarities due to phylogenetic and environmental factors (Ley et al., 2008; Ochman et al., 2010; Yildirim et al., 2010). Resembling the mid-level of Hinde (1976) social structure schemes, different social groups in the same population can exhibit distinct gut microbiota compositions associated with differences in diet or specific within-group patterns of relationships (Amato, 2013). Indeed, some of the available evidence suggests that gut microbiota similarity among individuals of the same primate social group (i.e., group membership) is frequently higher than among members of different social groups living in the same environment and even feeding on similar diets (Tung et al., 2015). Finally, a series of recent studies provide data suggesting that even within the same social group, interindividual similarity in gut microbiota may still be higher for dyads with more frequent or stronger social interactions (*sensu* Montiel-Castro et al., 2013).

Considering the correlates between gut microbiota and measurements derived from social structure, i.e., social interactions and relationships, we provide evidence of the tight link between sociality and gut microbiota composition, as suggested by Troyer (1984); Lombardo (2008), and Montiel-Castro et al. (2013). Within this context, and supporting our predictions, baboons with grooming partners had more similar communities of gut bacteria than individuals who rarely groomed each other, even when controlling for kinship and diet similarity between grooming partners (Tung et al., 2015; Grieneisen et al., 2017). Similarly, sifakas groups with denser grooming networks showed more homogeneous gut microbial compositions. Within social groups, adults and more gregarious individuals that scent-mark frequently harbored the greatest microbial diversity (Perofsky et al., 2017). Grooming- and huddling-based association indices were both positively correlated with microbiota similarity among red-bellied lemurs (Raulo et al., 2018) and rhesus monkeys (Balasubramaniam et al., 2019). However, in this case, contrary to expectations, grooming- and huddling-based individual sociality was negatively associated with gut microbial alpha diversity (Raulo et al., 2018). These results highlight the importance of social interactions and social relationships for microbial exchange between conspecifics. However, in species with low rates of grooming or social contact the repertoire of social interactions is more restricted, and thus, we should consider the role of other social interactions.

Concerning the influence of kinship on gut microbiota composition, we propose that some degree of microbial similarity between females with matrilines could be based on kinship. For example, women from Fiji, compared to men, harbored bacterial strains more closely related to their familial contacts (Brito et al., 2019). Conversely, primatological studies suggest that patterns of kin relatedness may not have a significant role as predictors of gut microbiota similarity in primates, which support the findings of Rothschild et al. (2018). In this context, in social groups of baboons, characterized by female philopatry and composed by several matrilines (Silk et al., 2006), genetic relatedness did not correlate significantly with similarity in microbial communities after controlling for group membership (Tung et al., 2015; Grieneisen et al., 2017). A similar result was observed in chimpanzees, Sifakas, and howler monkeys (Degnan et al., 2012; Moeller et al., 2016; Amato et al., 2017; Perofsky et al., 2017). Within social groups of Sifakas, individuals belonging to the same maternal line did not share, on average, more bacterial phylotypes compared to related individuals belonging to different maternal lines or unrelated group members, suggesting that kinship does not drive the compositional homogeneity found among conspecifics (Perofsky et al., 2017). In arboreal and small social primate groups, and after controlling for time spent in contact and in close proximity, Amato and colleagues observed in howler monkeys that closely related individuals had less similar gut microbial communities than non-related individuals. Moreover, compared to other adult-juvenile dyads, mother-offspring dyads did not have more similar gut microbial communities (Amato et al., 2017). Nonetheless, more studies are needed to clarify these results and, particularly, evaluate whether the size of matrilines could be hiding the influence of kinship on gut microbial similarity within matrilines.

### Mating and care systems

Lastly, the empirical evidence linking mating and care systems with gut microbial composition is scarce. Whereas certain sexually anatomic characteristics and courtship and mating behaviors influence the reproductive microbiome diversity in primates (Yildirim et al., 2014) and other taxa (MacManes, 2011; White et al., 2011), their influences on gut microbiota composition have not been tested yet. With respect to care systems, we found that, in addition to maternal vertical transmission during birth and lactation, parental care style, and non-parental interactions shape the early life assembly of gut microbiota amongst weaned rhesus macaques (Ardeshir et al., 2014; Amaral et al., 2017; Dettmer et al., 2019). Nonetheless, this also needs to be tested in wild settings to distinguish the influences on gut microbiota of parental and non-parental behavior from other kind of social behaviors. Therefore, as Kappeler (2019) suggests, we should consider the four components (social organization, social structure, and mating and care system) to characterize the social complexity of animal groups and explain significant variations across studies while aiming at understanding the particularities of microbial exchange based on social pathways.

## Social group size, neocortex size, and gut microbial diversity: Quantitative evidence in primates

One of the main conclusions derived from the “Social Brain Hypothesis” (Dunbar, 1998) is that across species’ evolution, cognitive capacities indexed by brain size or neocortex size have increased under the pressure of supplying the cognitive resources required to follow and participate in a greater diversity of possible behavioral interactions taking place in large group sizes (Dunbar and Shultz, 2021). However, comparative evidence suggests that greater cognitive capacities may also produce reductions in the number of social partners, associated to intense and even pair-bonded social relationships (Dunbar and Shultz, 2007). Furthermore, stronger social partner selection based on greater cognitive ‘power’ and within-group microbial exchange may serve as part of the behavioral immune system, limiting the range of interactions found within social groups (Fincher and Thornhill, 2008). In this context, if greater neocortex size is positively correlated with social group size (i.e., a simple measure of social complexity) across different species and the social microbiome increases its diversity in proportion to increments in social group size, then we can also predict that increased cognitive capacity to select social partners will reduce both group size and the diversity of the social microbiome.

Thus, to test for variations in microbial diversity, based on group size and neocortex size, we developed a metanalysis based on reported values from different studies, including the following variables: species’ specific Average group size, Neocortex size, and Body weight (Supplementary Table 1 and Supplementary Information) as predictors of Maximum number of observed microbial features (measured as the maximum number of operational taxonomic units, OTUs, or amplicon sequence variants, ASVs). Given that body weight may in turn correlate with gut length (Hounnou et al., 2002) (i.e., larger guts could provide more extensive areas for a greater diversity of gut microbial communities to establish) and with larger brain size, we included body weight as another predictor in this analysis. Therefore, through multiple linear regressions, we detected significant linear relationships (Methods described in Supplementary Information). The significant model (*R*^2^ = 0.42; Adj *R*^2^ = 0.40; *F*(3,30) = 20.70, *P* < 0.001), found that species’ Average group size was the single significant predictor (Table 1) of Maximum observed features. This analysis suggests, as expected, that larger group sizes, i.e., the simplest indicator of a group’s social complexity, predict a greater microbial diversity in the primate gut microbiota (Figure 3).

**TABLE 1:** Best-fit final stepwise linear regression (backward) model with Maximum number of observed microbial features (OTUs or ASVs) as dependent variable.

	Predictor	B	SE	StB	t	*P*	Tolerance	VIF
**(Constant)**		389.6	336.65		1.16	0.26		
	Average group size*	26.13	5.74	0.64	4.55	<0.001	1.0	1.0

*R^2^ = 0.42; Adj R^2^ = 0.40; F(3,30) = 20.70, P < 0.001; N = 31.

## Potential biochemical and molecular mechanisms that gut bacteria could employ to tolerate the external environments

Metacommunity theory applied to host-microbial systems take into account the feedback between the host-as-patch and its microbial communities (Miller et al., 2018). As part of this feedback, the microbiome affects the condition of the host through, for instance, behavioral changes, and the patch (host) can ‘manipulate’ the composition of the microbial community owing to its dynamic’s nature (Miller et al., 2018). This implies, on the one hand, the understanding of the role that the microbiome could play in the evolution of social behavior. For now, mathematical models suggest that the microbiome is key to explain the evolution of complex social behaviors, such as altruism, cooperation, and parental care (Lewin-Epstein et al., 2017; Gurevich et al., 2020; Lewin-Epstein and Hadany, 2020). On the other hand, several hosts forming a social group can modify their social microbiome, as well as microbial transmission routes (Miller et al., 2018), by making shared or unshared decisions for accessing to resources (e.g., beneficial endosymbionts). In turn, these effects may affect metacommunity coexistence processes, such as competition-colonization trade-off in a patch dynamic system (Miller et al., 2018). Therefore, talking about the ‘social microbiome’ inherently refers to successful microbial transmission between partners or conspecifics by social processes at different organizational levels (Sarkar et al., 2020). Nonetheless, for this to occur it is crucial to be aware of the specific molecular and behavioral mechanisms allowing commensal gut bacteria to be transferred from one host to another, for instance, through a fecal-oral route of transmission (Browne et al., 2017). This transmission route requires that gut microorganisms survive and persist in stressful conditions (external and along a new gastrointestinal tract) while remaining viable to efficiently colonize the gut of other hosts.

Most often, gut microbes are deposited in the external environment as feces, which implies changes in bacterial metabolism and/or cellular architecture to maximize survival, avoiding the toxic effects of external conditions such as atmospheric oxygen, ultraviolet radiation, adverse temperatures and scarce or limited nutrients (Browne et al., 2017). Atmospheric oxygen availability is often mentioned as a detrimental factor for DNA and proteins of anaerobic bacteria, although many gut anaerobe bacteria show varying levels of tolerance (from minutes—*Roseburia* spp.—to hours—*Bifidobacterium* spp—or even days—*E. coli*—) being able to maintain metabolism using oxygen as the terminal electron acceptor (García-Bayona et al., 2020). For instance, *Bacteroides* species are metabolically and energetically robust and continue growing at 0.10 to 0.14 oxygen concentrations (Baughn and Malamy, 2004; García-Bayona et al., 2020), whereas *Faecalibacterium pausnitzii* relies on an extracellular flavin-thiol electron shuttle to survive in the presence of oxygen (Khan et al., 2012). *Akkermansia muciniphila* uses the cytochrome *bd* complex coupled to an unidentified NADH dehydrogenase to use oxygen as a final electron acceptor, which shifts metabolic process toward a higher acetate-to-propionate ratio, resulting in more ATP and NADH, eventually leading to a slightly increased yield and growth rate (Ouwerkerk et al., 2016).

Another strategy to preserve microbial DNA integrity, usually found in phylogenetically-diverse and human-associated pathogens (one that may be widespread in gut microbiota), is a viable, but non-culturable state which is a form of bacterial dormancy involving decrements in metabolic activity and the generation of a strengthened cell wall achieved by modifications to the peptidoglycan structure (Browne et al., 2017). A similar strategy to cope with environmental stress is the development of resilient, metabolically dormant structures known as spores, with integrity and fecundity conditions maintained by binding of DNA to small acid-soluble proteins in the spore core, in turn surrounded by several endurable protein layers with low permeability and high levels of peptidoglycan (Browne et al., 2017). Many spore-forming gut bacteria, such as members of Firmicutes (Browne et al., 2021), are known to promote homeostasis through the induction of immunomodulatory regulatory T cells, potentially representing up to 30% of the microbial abundance in the gut (Browne et al., 2017). However, spore-forming bacteria are less abundant within individuals but more prevalent in the human population compared to non-spore-forming bacteria (Hildebrand et al., 2021). Spore formation is associated, at least in gut Firmicutes, with features of host-adaptation such as genome reduction and specialized metabolic capabilities (Seedorf et al., 2014; Browne et al., 2021; Hildebrand et al., 2021). This suggests that at least for gut Firmicutes, host adaptation is an evolutionary trade-off between transmission and colonization abundance (Browne et al., 2021).

Because of the gregarious nature of bacteria, they are commonly found forming highly structured multispecies communities, known as microbial biofilms (Stoodley et al., 2002; Nadell et al., 2008) where individual survival strategies may co-occur. From the perspective of the principles established by sociobiology, social behavior is common among microorganisms (Crespi, 2001; Foster, 2010) and beneficial microbes are capable of cooperating with the host’s intrinsic immune capabilities to diminish pathogen colonization (Sassone-Corsi and Raffatellu, 2015). In biofilms, cooperation occurs at a local level, yet strong conflicts can still arise among multiple species and strains. Moreover, spontaneous mutation generates conflicts even within bacterial biofilms initiated by genetically identical cells (Nadell et al., 2008). Therefore, biofilm formation may be conceived as a result from a balance between bacteria cooperation and competition (Nadell et al., 2008).

Biofilm’s bacteria populations rearrange nucleotides, sugar, amino acids, and energy metabolism (Caro-Astorga et al., 2020). These modifications to metabolic arrangement coexist with spore formation, adhesion, motility, synthesis of extracellular polymers, activation of the ROS detoxification, machinery, and production of metabolites (Nadell et al., 2008; Pascoe et al., 2015; Caro-Astorga et al., 2020). Motility, adherence, and biofilm formation are behaviors that are often regulated by quorum sensing, a form of cell-to-cell communication in which small chemical signals, known as autoinducers, are secreted and accumulate as bacteria multiply (Thompson et al., 2016). Moreover, structural adaptations and interrelationships are made possible by the expression of sets of genes that result in segregated subpopulations with different phenotypes and physiological properties (Nadell et al., 2008; Pascoe et al., 2015; Ch’ng et al., 2019). Therefore, the characteristics of bacteria’s biofilms often differ as culture condition changed (Nadell et al., 2008) and biofilm formation represents a protected growth-mode allowing microorganisms to survive in hostile environments and dispersing into new niches (Hall-Stoodley et al., 2004; Pascoe et al., 2015). Such colonization involves a passage through the gastrointestinal tract, the establishment of a niche in the intestinal environment, the use of available nutrients, and replication to levels ensuring stability and survival (Browne et al., 2017).

Essentially, the transmission, and subsequent colonization of oral microbes from phylogenetically diverse microbial taxa into the gut may be a constant process in healthy individuals. Approximately one in three oral microbial cells pass through the digestive tract to settle in the gut, accounting for at least 2% of the classifiable microbial abundance in feces (Schmidt et al., 2019). Although a given host may select commensal bacteria by modulation of the intestinal environment by genetic (e.g., expression of fucosyltransferase 2 gene) and immunological (e.g., PSA signals through TLR2 directly on Foxp3^+^ regulatory T cells) responses (Coyne et al., 2008; Browne et al., 2017), beneficial microorganisms find an assortment of harsh environment conditions during their passage through gastrointestinal tract. These include digestive enzymes, acid pH in the stomach, defensins, and high concentrations of bile salts in the intestine (González-Rodríguez et al., 2013). Nonetheless, Bifidobacteria survive the gastrointestinal transit using stress response mechanisms (such as overproduction of subunit of the F0-F1-ATPase, production of exopolysaccharides and efflux pumps) and specific molecules (extracellular proteins like BopA) involved in adhesion and colonization factors, as well as by taking advantage of specific energy recruitment pathways (González-Rodríguez et al., 2013).

During intestinal colonization the regulation of surface architecture appears to be critical for establishing the commensal association of *Bacteroides fragilis* with its mammalian host (Liu et al., 2008). This microorganism presents a class of polysaccharide utilization loci (commensal colonization factors, *ccf*) that is highly induced during gut colonization and bacterial growth in mucin O-glycans (Lee et al., 2013; Donaldson et al., 2018). Furthermore, such *ccf* locus regulates the expression of specific capsular polysaccharides to attract IgA binding allowing for mucosal colonization (Donaldson et al., 2018). Lastly, one quorum sensing molecule, Autoinducer-2, upregulates the adherence to epithelial cells in *Actinobacillus pleuropneumoniae* and increases the expression of motility genes for several strains of *E. coli*. Therefore, Autoinducer-2, which is produced by multiple bacterial species (e.g., *Bacteroides* spp., *Ruminococcus* spp., *Lactobacillus* spp.) likely regulate bacterial behavior and community dynamics in the microbiota (Thompson et al., 2016). In summary, both host and gut microbes are supplied with selective and/or specific mechanisms, simultaneously operating to avoid pathogen spread and encourage successful transmission and colonization of beneficial endosymbionts. Therefore, these mechanisms must also be considered as part of the feedback between the host-as-patch and its microbial communities.

## Conclusion

There is increasing evidence of the pivotal role that “good” and “bad” microorganisms play on host health. However, only the reality of the COVID-19 pandemic, starting more than two years ago, has helped us to conceive the actual implications of microbial transmission mediated by social contact within a highly connected world. Understanding this process has become a race for solutions that help to maintain or improve our physiological, immunological, and neurological health, which may be understood as the ‘equilibrium state’ between beneficial and detrimental forces. On the one hand, research on socially microbial transmission is focused on avoiding or slowing the spread of infectious diseases caused by pathogens. On the other hand, studies suggest that social transmission of commensal microorganisms should be fostered to preserve their health-related benefits. In this context, we propose a tight connection between gut microbiota and social complexity within primate groups. We outline predictions concerning how social organization, social structure, as well as mating and care systems may shape the gut microbiota composition at group level. Primate complexity is extensive and while some components receive more attention, others are not appropriately attended yet, like parental care, which seems to be a key aspect for immune and neuronal development in early-life when gut microorganisms play important priming—programming roles. More research is needed to understand the interplay between gut microbial communities and social behavior. An important conclusion is that, since social complexity represents an emergent property of individual social interactions, health may be favored by improving our own social (microbiota-exchange) connections network. Nonetheless, the recent experience suggests extreme care and analyses of the conditions regarding how certain microorganisms may be transmitted by local social behavior (at group level) with effects scaling up to a global order (at population level), with possibly serious public health consequences. Therefore, we consider that a profound and better understanding of the interplay between transmission of microorganisms and social interactions is necessary to distinguish between beneficial and pathogenic capabilities of socio-microbial exchange.

## Author contributions

BP-G and AM-C organized the database, performed the statistical analysis, and wrote the first draft of the manuscript. All authors contributed to the conception and design of the study, manuscript revision, read, and approved the submitted version.
