# Supplementary material for: Social complexity as a driving force of gut microbiota exchange among conspecific hosts in non-human primates
Source: Front Integr Neurosci. 2022 Aug 30;16:876849. doi: 10.3389/fnint.2022.876849 (PMC9468716; doi:10.3389/fnint.2022.876849)
Supplement: Supplementary file 1 [file Data_Sheet_1.docx]

**Social complexity as a driving force of gut microbiota exchange among conspecific hosts in nonhuman primates**

Braulio Pinacho-Guendulain, Augusto Jacobo Montiel-Castro, Gabriel Ramos-Fernández, Gustavo Pacheco-López

**Supplementary Information**

Methods

As search methods and inclusion criteria we used Google Scholar to identify any primatological study that, first, related the gut microbiota with different traits of social components and, secondly, reported any measure of the gut microbiota composition: such as total, average, minimum and maximum number of observed features (operational taxonomic units, OTUs, or amplicon sequence variants, ASVs). We found a total of 36 studies of 20 primate species, including humans, published between 2010 and 2021. Most of these were cross-sectional studies (29 papers), with a few longitudinal (5 studies), and only 2 studies including both cross-sectional and longitudinal data. Moreover, thirty studies were conducted with wild primates, 3 with captive subjects, and only 2 studies included both wild and captive individuals.

Of this sample, at least 17 studies concerned the interplay between gut microbiota and host sociality, including 12 primate species. Regarding the association between gut microbiota and social complexity, we employed microbial data from 17 studies conducted in 36 wild groups of 15 primate species (see Table S1). Data of neocortex ratio were obtained from Stephan et al. (1981), Dunbar (1992), and Kudo and Dunbar (2001). Neocortex size of *Piliocolobus tephrosceles* was found in Lehmann et al. (2007). Information of body weight and average group size were obtained from (DeCasien et al. 2017). Lastly, average group size for humans was determined as described by Aiello and Dunbar (1993).

Based on these data, we used multiple linear regressions to test for (i) a quantitative influence of social group size, as a simple measure of social complexity, upon the gut microbiota, and (ii) a possible quantitative influence of neocortex ratio upon the gut microbiota. The particular method chosen was a stepwise, backward multiple linear regression, considering it preferable to the forward method due to suppressor effects and lower risks of making a Type II error (Field 2013). In this method, all predictors are first included in the model and are then excluded based on the significance of their contribution. For each predictor the significance value of a t-test is compared against a removal criterion (here set to P ≤ 0.050). When the predictor does not make a significant contribution, it is removed and the model is re-calculated, testing the contribution of each remaining predictor (Field 2013). Additionally, case-wise diagnostics were used, considering data as outliers beyond Mean ± 2SD. All statistical analyses were carried out using SPSS 21.0.

Table A1 - Excluded variables for the best-fit final stepwise linear regression (backward) model with Maximum observed features (OTUs or ASVs) as the dependent variable.

|  | Beta in | t | P |  | Partial  correlation | Tolerance | VIF |
| --- | --- | --- | --- | --- | --- | --- | --- |
| Body weight (g) | 0.079 | 0.546 | 0.590 |  | 0.103 | 0.991 | 1.009 |
| Neocortex ratio | 0.259 | 1.346 | 0.189 |  | 0.246 | 0.530 | 1.885 |

Table A2 – Casewise diagnostics for the best-fit final stepwise linear regression (backward) model with Maximum observed features (OTUs or ASVs) as the dependent variable.

| Case number | Species | Std residual | Maximum observed features | Predicted value | Residual |
| --- | --- | --- | --- | --- | --- |
| 16 | *Homo sapiens* | 4.054 | 10079 | 4257.51 | 5821.493 |

Table A3 – Residual statistics for the best-fit final stepwise linear regression (backward) model with Maximum observed features (OTUs or ASVs) as the dependent variable.

|  | Minimum | Maximum | Mean | Std. Deviation | N |
| --- | --- | --- | --- | --- | --- |
| Predicted value | 544.58 | 4343.75 | 1373.97 | 1192.752 | 31 |
| Residual | -2613.751 | 5821.493 | 0.0 | 1411.863 | 31 |
| Std. Predicted value | -0.695 | 2.490 | 0.0 | 1.0 | 31 |
| Std. Residual | -1.820 | 4.054 | 0.0 | 0.983 | 31 |


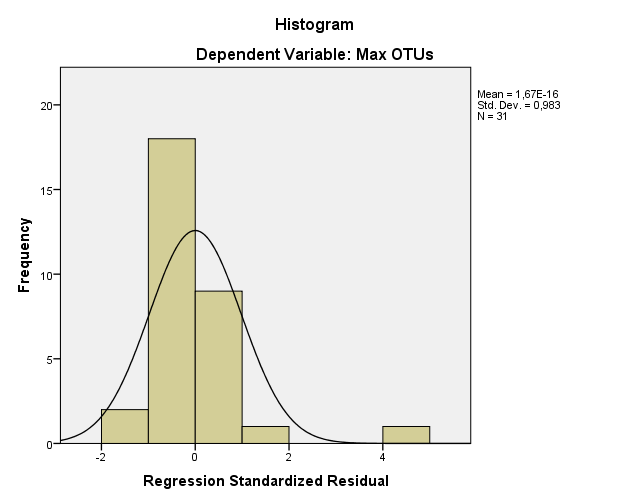


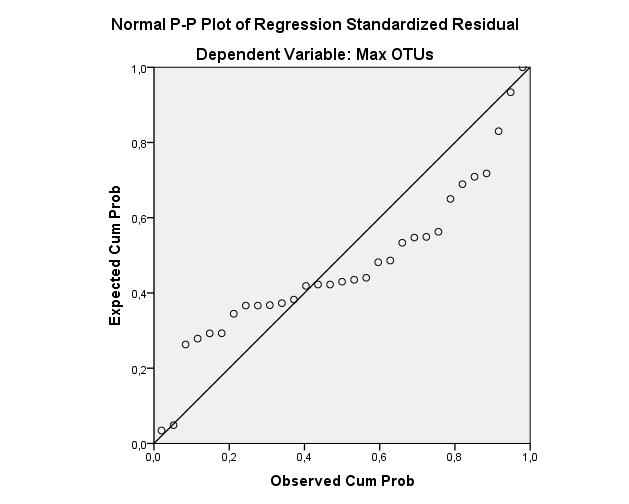


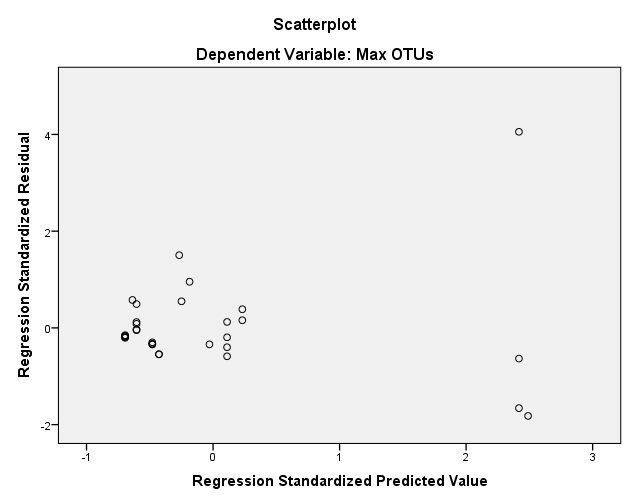


Table S1. Social group size, neocortex ratio and gut microbial diversity for 36 social primate groups.

| Species | Neocortex ratio | Body weight (g) | Average group size | Observed Features | | | | | Reference |
| --- | --- | --- | --- | --- | --- | --- | --- | --- | --- |
|  |  |  |  | **Total** | **Average** | **Standard deviation** | **Minimum** | **Maximum** |  |
| *Alouatta pigra* | 2.15 | 8940 | 5.93 |  | 262 | 48 | 182 | 329 | Amato et al. (2017) |
| *Alouatta pigra* | 2.15 | 8940 | 5.93 |  | 247 | 15 | 231 | 264 | Amato et al. (2017) |
| *Alouatta pigra* | 2.15 | 8940 | 5.93 |  | 244 | 47 | 181 | 291 | Amato et al. (2017) |
| *Alouatta pigra* | 2.15 | 8940 | 5.93 |  | 289 | 21 | 255 | 310 | Amato et al. (2017) |
| *Alouatta pigra* | 2.15 | 8940 | 5.93 |  | 223 | 28 | 181 | 250 | Amato et al. (2017) |
| *Cercopithecus ascanius* | 2.27 | 4157 | 26.29 |  | 1666 |  | 1306 | 1867 | Yildirim et al. (2010) |
| *Colobus guereza* | 2.32 | 10052 | 8.6 |  | 1295 |  | 1072 | 1442 | Yildirim et al. (2010) |
| *Gorilla gorilla* | 2.65 | 121327 | 10.05 | 2644 | 617 | 103 | 495 | 823 | Gomez et al. (2015) |
| *Gorilla gorilla* | 2.65 | 121327 | 10.05 | 2312 | 576 | 78 | 476 | 773 | Gomez et al. (2015) |
| *Gorilla gorilla* | 2.65 | 121327 | 10.05 | 1433 | 530 | 42 | 450 | 586 | Gomez et al. (2015) |
| *Gorilla gorilla* | 2.65 | 121327 | 10.05 | 1784 | 533 | 66 | 443 | 603 | Gomez et al. (2015) |
| *Gorilla gorilla* | 2.65 | 121327 | 10.05 |  | 753 | 212 | 542 | 1361 | Narat et al. (2020) |
| *Homo sapiens* | 4.1 | 65078 | 148 | 1235 | 186 | 51 |  |  | Yang et al. (2020) |
| *Homo sapiens* | 4.1 | 65078 | 148 |  | 1427 | 847 | 51 | 3347 | Das et al. (2018) |
| *Homo sapiens* | 4.1 | 65078 | 148 |  | 1212 | 265 | 771 | 1876 | Das et al. (2018) |
| *Homo sapiens* | 4.1 | 65078 | 148 |  | 1770 | 1831 | 532 | 10079 | Das et al. (2018) |
| *Lemur catta* | 1.18 | 2380 | 15.75 | 1821 |  |  |  |  | Fogel (2015) |
| *Lemur catta* | 1.18 | 2380 | 15.75 | 501 | 293 | 22 | 263 | 311 | Bennett et al. (2016) |
| *Lemur catta* | 1.18 | 2380 | 15.75 | 854 | 280 | 49 | 201 | 372 | Bennett et al. (2016) |
| *Lemur catta* | 1.18 | 2380 | 15.75 | 765 | 288 | 31 | 255 | 335 | Bennett et al. (2016) |
| *Lemur catta* | 1.18 | 2380 | 15.75 | 610 | 273 | 39 | 208 | 316 | Bennett et al. (2016) |
| *Macaca mulatta* | 2.6 | 6472 | 36.36 | 1770 | 637 | 134 | 153 | 850 | Li et al. (2021) |
| *Macaca thibetana* | 2.6 | 7190 | 25.46 |  | 1241 | 408 | 754 | 3213 | Sun et al. (2016) |
| *Pan troglodytes* | 3.22 | 48328 | 42.71 |  | 949 | 265 | 518 | 1683 | Degnan et al. (2012) |
| *Pan troglodytes* | 3.22 | 48328 | 42.71 |  | 872 | 199 | 582 | 1225 | Degnan et al. (2012) |
| *Pan troglodytes* | 3.22 | 48328 | 42.71 |  | 497 | 60 | 381 | 663 | Moeller et al. (2016) |
| *Pan troglodytes* | 3.22 | 48328 | 42.71 |  | 792 | 136 | 585 | 932 | Narat et al. (2020) |
| *Papio cynocephalus* | 2.68 | 14291 | 48.21 | 11547 | 1440 | 226 | 958 | 1876 | Grieneisen et al. (2017) |
| *Papio cynocephalus* | 2.68 | 14291 | 48.21 | 14850 | 1638 | 307 | 704 | 2203 | Grieneisen et al. (2017) |
| *Piliocolobus tephrosceles* | 2.22 | 8105 | 29.18 |  | 2003 |  | 1697 | 2522 | Yildirim et al. (2010) |
| *Propithecus verreauxi* | 1.1 | 3389 | 5.92 | 1721 |  |  |  |  | Fogel (2015) |
| *Pygathrix nemaeus* | 2.05 | 9250 | 21.45 |  | 4231 | 584 |  |  | Clayton et al. (2018) |
| *Sapajus nigritus* | 2.25 | 2662 | 18.14 | 84 | 75 | 9 | 59 | 81 | Grassotti et al. (2021) |
| *Sapajus nigritus* | 2.25 | 2662 | 18.14 | 83 | 74 | 11 | 54 | 80 | Grassotti et al. (2021) |
| *Theropithecus gelada* | 2.55 | 14778 | 151.3 | 1624 |  |  |  |  | Trosvik et al. (2018) |
| *Theropithecus gelada* | 2.55 | 14778 | 151.3 | 3295 | 813 | 243 | 92 | 1730 | Baniel et al. (2021) |

References

Aiello, Leslie C and Dunbar, Robin IM (1993), 'Neocortex size, group size, and the evolution of language', *Current anthropology,* 34 (2), 184-93.

Amato, Katherine R., et al. (2017), 'Patterns in Gut Microbiota Similarity Associated with Degree of Sociality among Sex Classes of a Neotropical Primate', *Microbial Ecology,* 74 (1), 250-58.

Baniel, Alice, et al. (2021), 'Seasonal shifts in the gut microbiome indicate plastic responses to diet in wild geladas', *Microbiome,* 9 (1), 26.

Bennett, Genevieve, et al. (2016), 'Host age, social group, and habitat type influence the gut microbiota of wild ring-tailed lemurs (Lemur catta)', *American Journal of Primatology,* 78.

Clayton, Jonathan B., et al. (2018), 'Associations Between Nutrition, Gut Microbiome, and Health in A Novel Nonhuman Primate Model', *Scientific Reports,* 8 (1), 11159.

Das, Bhabatosh, et al. (2018), 'Analysis of the Gut Microbiome of Rural and Urban Healthy Indians Living in Sea Level and High Altitude Areas', *Scientific Reports,* 8 (1), 10104.

DeCasien, Alex R., Williams, Scott A., and Higham, James P. (2017), 'Primate brain size is predicted by diet but not sociality', *Nature Ecology &Amp; Evolution,* 1, 0112.

Degnan, Patrick H., et al. (2012), 'Factors associated with the diversification of the gut microbial communities within chimpanzees from Gombe National Park', *Proceedings of the National Academy of Sciences,* 109 (32), 13034-39.

Dunbar, R. I. M. (1992), 'Neocortex size as a constraint on group size in primates', *Journal of Human Evolution,* 22 (6), 469-93.

Field, Andy (2013), *Discovering statistics using IBM SPSS statistics* (sage).

Fogel, Andrew T (2015), 'The gut microbiome of wild lemurs: a comparison of sympatric Lemur catta and Propithecus verreauxi', *Folia Primatologica,* 86 (1-2), 85-95.

Gomez, Andres, et al. (2015), 'Gut microbiome composition and metabolomic profiles of wild western lowland gorillas (Gorilla gorilla gorilla) reflect host ecology', *Molecular ecology,* 24 (10), 2551-65.

Grassotti, Tiela Trapp, et al. (2021), 'Fecal bacterial communities of wild black capuchin monkeys (Sapajus nigritus) from the Atlantic Forest biome in Southern Brazil are divergent from those of other non-human primates', *Current Research in Microbial Sciences,* 2, 100048.

Grieneisen, Laura E., et al. (2017), 'Group Living and Male Dispersal Predict the Core Gut Microbiome in Wild Baboons', *Integrative and Comparative Biology,* 57 (4), 770-85.

Kudo, H. and Dunbar, R. I. M. (2001), 'Neocortex size and social network size in primates', *Animal Behaviour,* 62 (4), 711-22.

Lehmann, J., Korstjens, A. H., and Dunbar, R. I. M. (2007), 'Group size, grooming and social cohesion in primates', *Animal Behaviour,* 74 (6), 1617-29.

Li, Yuhui, et al. (2021), 'Gut microbiota are associated with sex and age of host: Evidence from semi-provisioned rhesus macaques in southwest Guangxi, China', *Ecology and Evolution,* n/a (n/a).

Moeller, Andrew H., et al. (2016), 'Social behavior shapes the chimpanzee pan-microbiome', *Science Advances,* 2 (1).

Narat, Victor, et al. (2020), 'A multi-disciplinary comparison of great ape gut microbiota in a central African forest and European zoo', *Scientific Reports,* 10 (1), 19107.

Stephan, H., Frahm, H., and Baron, G. (1981), 'New and Revised Data on Volumes of Brain Structures in Insectivores and Primates', *Folia Primatologica,* 35 (1), 1-29.

Sun, Binghua, et al. (2016), 'Marked variation between winter and spring gut microbiota in free-ranging Tibetan Macaques (Macaca thibetana)', *Scientific Reports,* 6, 26035-35.

Trosvik, Pål, et al. (2018), 'Multilevel social structure and diet shape the gut microbiota of the gelada monkey, the only grazing primate', *Microbiome,* 6 (1), 84.

Yang, Jing, et al. (2020), 'Species-Level Analysis of Human Gut Microbiota With Metataxonomics', *Frontiers in Microbiology,* 11.

Yildirim, Suleyman, et al. (2010), 'Characterization of the Fecal Microbiome from Non-Human Wild Primates Reveals Species Specific Microbial Communities', *PLoS ONE,* 5 (11), e13963.
